# *Penaeus monodon* Interferon Regulatory Factor (*Pm*IRF) Activates IFNs and Antimicrobial Peptide Expression *via* a STING-Dependent DNA Sensing Pathway

**DOI:** 10.3389/fimmu.2021.818267

**Published:** 2022-01-10

**Authors:** Suthinee Soponpong, Piti Amparyup, Taro Kawai, Anchalee Tassanakajon

**Affiliations:** ^1^ Center of Excellence for Molecular Biology and Genomics of Shrimp, Department of Biochemistry, Faculty of Science, Chulalongkorn University, Bangkok, Thailand; ^2^ Marine Biotechnology Research Team, Integrative Aquaculture Biotechnology Research Group, National Center for Genetic Engineering and Biotechnology (BIOTEC), National Science and Technology Development Agency (NSTDA), Pathumthani, Thailand; ^3^ Center of Excellence for Marine Biotechnology, Department of Marine Science, Faculty of Science, Chulalongkorn University, Bangkok, Thailand; ^4^ Laboratory of Molecular Immunobiology, Division of Biological Science, Graduate School of Science and Technology, Nara Institute of Science and Technology (NAIST), Ikoma, Japan

**Keywords:** IRF, STING, DNA sensing pathway, antiviral response, interferon, shrimp immunity

## Abstract

Interferon regulatory factors (IRFs) are transcription factors found in both vertebrates and invertebrates that were recently identified and found to play an important role in antiviral immunity in black tiger shrimp *Penaeus monodon*. In this study, we investigated the mechanism by which *P. monodon* IRF (*Pm*IRF) regulates the immune-related genes downstream of the cytosolic DNA sensing pathway. Depletion of *Pm*IRF by double-stranded RNA-mediated gene silencing significantly reduced the mRNA expression levels of the IFN-like factors *Pm*Vago1, *Pm*Vago4, and *Pm*Vago5 and antilipopolysaccharide factor 6 (ALF*Pm*6*)* in shrimp. In human embryonic kidney (HEK293T) cells transfected with *Pm*IRF or co-transfected with DEAD-box polypeptide (*Pm*DDX41) and simulator of IFN genes (*Pm*STING) expression plasmids, the promoter activity of IFN-β, nuclear factor (NF-κB), and ALF*Pm*6 was synergistically enhanced following stimulation with the nucleic acid mimics deoxyadenylic–deoxythymidylic acid sodium salt [poly(dA:dT)] and high molecular weight (HMW) polyinosinic–polycytidylic acid [poly(I:C)]. Both nucleic acid mimics also significantly induced *Pm*STING, *Pm*IRF, and ALF*Pm*6 gene expression. Co-immunoprecipitation experiments showed that *Pm*IRF interacted with *Pm*STING in cells stimulated with poly(dA:dT). *Pm*STING, *Pm*IRF, and *Pm*DDX41 were localized in the cytoplasm of unstimulated HEK293T cells and *Pm*IRF and *Pm*DDX41 were translocated to the nucleus upon stimulation with the nucleic acid mimics while *Pm*STING remained in the cytoplasm. These results indicate that *Pm*IRF transduces the pathogen signal *via* the *Pm*DDX41–*Pm*STING DNA sensing pathway to induce downstream production of interferon-like molecules and antimicrobial peptides.

## Introduction

The innate immune system is the first line of host defense against invasive pathogens (1). Host pattern recognition receptors (PRRs) play a key role in recognizing nonself pathogen-associated molecular patterns (PAMPs). A number of PRRs have been described, including Toll-like receptor (TLR), nucleotide-binding oligomerization domain-like receptors (NLRs) and retinoic acid-inducible gene (RIG)-I–like receptors (RLRs) (2–4). Intracellular DNA sensors including DEAD-box polypeptide (DDX)41 and cyclic GMP–AMP synthase (cGAS) recognize cytoplasmic or nuclear pathogen-derived DNA (5–9).

DDX41 has been shown to directly bind to double-stranded (ds) DNA and stimulator of IFN genes (STING) protein *via* the DEAD domain and induce the activation of nuclear factor kappa B (NF-κB) and IFN production in mouse dendritic cells (10). In vertebrates, STING is an adaptor protein of the cytosolic DNA-sensing pathway that mediates the immune response to pathogens. Upon binding to foreign DNA or cyclic (c)GMP–AMP (a second messenger), DNA sensors activate STING (11, 12), leading to activation of TANK-binding kinase (TBK)1, which then phosphorylates STING and the downstream transcription factor IRF3 to induce the expression of type I IFNs (13) in the immune response to DNA viruses and tumors (14–16).

In vertebrates, cGAS activates STING which initiates a downstream signaling pathway leading to induce the expression of IFNs and other cytokines such as TNF-α and IL-6, and thereby trigger the host immune response. After viral and bacterial infection, dsDNA was released. Cytosolic DNA binds and activates cGAS, which catalyzes the synthesis of 2′3′-cGAMP from ATP and GTP. 2′3′-cGAMP binds to the ER adaptor STING, which traffics to the ER and the Golgi apparatus. STING then activates IKK and TBK1. TBK1 phosphorylates STING, which in turn recruits IRF3 for phosphorylation by TBK1. IRF3 then enters the nucleus, where it functions with NF-kB to synthesize the expression of IFNs (6, 17).

In *Drosophila*, the inhibitor of kappa B kinase (dIKKβ) and Relish genes were found to be induced by viral infection. The *Drosophila* STING ortholog (dSTING) participates in host defense against infection by picorna-like viruses, acting upstream of dIKKβ to regulate the expression of the antiviral factor Nazo (18). In Pacific white shrimp (*Litopenaeus vannamei*), *Lv*STING was shown to contribute to the innate immune response to infection by *Vibrio parahaemolyticus* (19). Similarly, *Pm*STING in black tiger shrimp *Penaeus monodon* which shared high sequence similarity to *Lv*STING (92%), was important for the antiviral innate response against white spot syndrome virus (WSSV) infection (20).

IRFs are a family of transcription factors involved in the antiviral response (21–23). To date, eleven IRFs (IRF-1 to IRF-11) have been identified in fish, all containing a highly conserved DNA-binding domain in the N-terminal region that recognizes a consensus sequence similar to the IFN-stimulated response element (ISRE) (24). IRF3 and IRF7 are activated by TLR3 and TLR4 signaling pathways, respectively, leading to IFN expression (25). IRF has been identified in *L. vannamei* (*Lv*IRF) (26) and more recently, in *P. monodon* (*Pm*IRF) (20). Like their vertebrate counterparts, *Lv*IRF and *Pm*IRF as well as *Pm*DDX41 and *Pm*STING are activated during virus infection (26,27). It was reported that *Lv*IRF mediated the activation of the ISRE-containing promoters in mammalian cells to regulate the expression of *Lv*Vago4 and *Lv*Vago5 genes, which encode a virus-activated secreted peptide that blocks virus infection *via* activation of the Janus kinase (JAK)–STAT signaling pathway (26).

Recently, we identified *Pm*DDX41, *Pm*STING and *Pm*IRF in shrimp *P. monodon*. *Pm*DDX41 plays an important role as a cytosolic DNA sensor which interacted with STING and triggered the IFNs and NF-κB signaling pathway to activate the innate immune response (27). While, *Pm*STING and *Pm*IRF play a key role in protecting shrimp from WSSV infection (20). Moreover, silencing of *Pm*DDX41 caused a decrease expression of *Pm*STING and *Pm*IRF (20). As the regulation of the cytosolic DNA-sensing pathways in shrimp is not fully understood, in this study, we further characterized the function of *Pm*IRF in antiviral innate immunity by identifying its downstream immune-related target genes. Nucleic acid mimics were used to stimulate the cells and investigate the innate immune response. Poly(dA:dT) and poly(I:C) are the synthetic analog of B form DNA and synthetic dsRNA polymer, respectively, thus representative of a DNA virus and RNA virus. It was found that *Pm*IRF, *Pm*DDX41, and *Pm*STING synergistically activated the IFN-β, NF-κB, and antilipopolysaccharide factor (ALF*Pm*6) gene promoters following stimulation with DNA mimics and that *Pm*IRF interacts with *Pm*STING in the cytoplasm and translocates to the nucleus to stimulate the expression and production of IFN-like molecules as part of the antiviral immune response in *P. monodon*.

## Materials and Methods

### Shrimp and Sample Preparation

Healthy black tiger shrimps (*P. monodon*; 3-5 g body weight) were provided by Charoen Pokphand Foods in Chanthaburi province, Thailand, and maintained in aerated seawater (20 ppt) at 28°C for 1 week prior to experiments. Shrimp samples were screened for pathogen-free including WSSV, YHV, EHP and *Vibrio parahaemolyticus* AHPND by PCR before used in the experiment. To determine the expression level of the *Pm*IRF transcript, intestine from triplicate groups of 3 shrimps each were separately collected as previously described (28). All samples were stored at -80°C until RNA extraction. This study was conducted under the ethical principles and guidelines according to the animal use protocol approved by Chulalongkorn University Animal Care and Use Committee (CU-ACUC).

### Total RNA Extraction and Reverse Transcription

Shrimp intestine was homogenized in GENEzol (Geneaid, New Taipei City, Taiwan) and total RNA was isolated according to the manufacturer’s protocol and treated with DNaseI (New England Biolabs, Ipswich, MA, USA) to destroy contaminating DNA. First-strand cDNA was synthesized using the RevertAid First Strand cDNA Synthesis Kit (Thermo Fisher Scientific, Waltham, MA, USA) and stored at -20°C until used for qRT-PCR.

### Double-Stranded RNA Preparation

To prepare dsRNA specifically targeting *Pm*IRF, DNA fragments of the *Pm*IRF (614 bp) gene were amplified by PCR using specific primers (*Pm*IRFi-F1 and *Pm*IRFi-R1) designed using the Primer Premier 5 program (PREMIER Biosoft, Palo Alto, CA, USA) (**Table 1**). *In vitro* transcription with T7 RNA polymerase was performed to obtain sense and antisense RNA strands. Sense and antisense DNA templates containing the T7 promoter RNA polymerase sequence at the 5’ end of each strand were generated by PCR using oligonucleotide primers containing the sequence at the 5’ end (*Pm*IRFi-T7F1 and *Pm*IRFi-T7R1) (**Table 1**). For the negative control dsRNA, the GFP gene was amplified from the pEGFP-1 plasmid (Clontech, Mountain View, CA, USA) (29). The T7 RiboMAX Express Large Scale RNA Production System (Promega, Madison, WI, USA) was used to synthesize RNA by *in vitro* transcription according to the manufacturer’s protocol. The quality of the dsRNA was verified by agarose gel electrophoresis and quantification was performed by spectrophotometry.

**Table 1:** Primers used in experiments.

Primer Purpose and Name	Sequence (5’to3’)
RNAi	
*Pm*IRFi-F1	GCTGCTCTGTTTCGCTATTGGG
*Pm*IRFi-R1	GGGTCGCTCTTGGCGGTCGGAT
*Pm*IRFi-T7F1	GGATCCTAATACGACTCACTATAGGGCTGCTCTGTTTCGCTATTGGG
*Pm*IRFi-T7R1	GGATCCTAATACGACTCACTATAGGGGGTCGCTCTTGGCGGTCGGAT
Transcription study	
*Pm*IRF-F	CTACGACATATCCTGTACGG
*Pm*IRF-R	GGTAGTAATCGTAGCCAGCT
*Pm*STING-F	CATGCGCCTCTGGTCACTA
*Pm*STING-R	CTCCATCACATCCAAGGCG
EF1-α-F	GGTGCTGGACAAGCTGAAGGC
EF1-α-R	CGTTCCGGTGATCATGTTCTTGATG
*Pm*Vago1-F	GAACACACCCCAGTGCACTGGT
*Pm*Vago1-R	ATGGAGCTTGTTCCCCTTCTGTG
*Pm*Vago2-F	CAACTATGAGGAGGGATGGGCAC
*Pm*Vago2-R	GTCCTGTTGTTCCTCGCTGTCG
*Pm*Vago3-F	GCACGAGGCAGTTCAGTGTCCT
*Pm*Vago3-R	CTC GGG CAG CAT TTC GGA TGA G
*Pm*Vago4-F	ACTCCTCTCCCTTCAGGGCATC
*Pm*Vago4-R	TGGCAGGAACTTCTCTCGCTGC
*Pm*Vago5-F	AGAAGCATTTAGGCTCAGGGCAG
*Pm*Vago5-R	GATGGCCAGAGTTATTGTGACGC
ALF*Pm*3-F	CCCACAGTGCCAGGCTCAA
ALF*Pm*3-R	TGCTGGCTTCTCCTCTGATG
ALF*Pm*6-F	AGTCAGCGTTTAGAGAGGTT
ALF*Pm*6-R	GCTCGAACTCTCCACTCTC
Crustin*Pm*1-F	CTGCTGCGAGTCAAGGTATG
Crustin*Pm*1-R	AGGTACTGGCTGCTCTACTG
Crustin*Pm*7-F	GGCATGGTGGCGTTGTTCCT
Crustin*Pm*7-R	TGTCGGAGCCGAAGCAGTCA
*Pm*PEN3-F	GGTCTTCCTGGCCTCCTTCG
*Pm*PEN3-R	TTTGCATCACAACAACGTCCTA
*Pm*PEN5-F	ATCCCGACCTATTAGTACTC
*Pm*PEN5-R	TTATCCTTTCAATGCAGAACAA
Protein expression in HEK293T cells	
FlagCMV5_*Pm*IRF_SalI_F	CGCGTCGACGTCGGCATGCCGCCATCTTTCACCG
FlagCMV5_*Pm*IRF_BamHI_R	CGCGGATCCGCGTTATCTCATTAGCATATAACTGT
Myc_*Pm*IRF_BamHI_F	ATAGGATCCAAAATGCCGCCATCTTTCACCG
Myc_*Pm*IRF_NheI_R	CTAGCTAGCTAGTCTCATTAGCATATAACTGT
FlagCMV5_*Pm*STING_HindIII_F	CCCAAGCTTGGGATGAAGGGAGACGAGCTGG
FlagCMV5_*Pm*STING_SalI_R	CGCGTCGACGTCGGCTCACTTCCGTTCCGTCATTT
Myc_*Pm*STING_HindIII_F	CCCAAGCTTGGGATGAAGGGAGACGAGCTGG
Myc_*Pm*STING_XhoI_R	CCGCTCGAGCGGCTTCCGTTCCGTCATTTCGT

### Gene Knockdown by RNA Interference (RNAi)

*Pm*IRF or GFP (control) dsRNA was injected into juvenile shrimp (3–5 g, fresh weight) using a 0.5-ml insulin syringe with a 29-gauge needle as previously described (29). Shrimp were injected with 25 ul of *Pm*IRF dsRNA (5 μg/g) diluted in 150mM NaCl. and delivered by intramuscular injection into the third abdominal segment of each shrimp. NaCl (150 mM) was injected as a control for handling- and injection-induced mortality. After 24 h, shrimp intestine was collected for total RNA extraction and first-strand cDNA was synthesized from 200 ng of total RNA as described above.

The efficiency of *Pm*IRF knockdown was analyzed by qRT-PCR using specific primers for *Pm*IRF (**Table 1**). A fragment of the elongation factor (EF)1-α gene was amplified in a separate tube and served as an internal control for normalization of expression levels. The PCR reactions and thermal cycling conditions were as previously reported (Soponpoong et al., 2008). In brief, the PCR reaction was performed in 10-µl reaction volume, containing 0.5 µl of intestine cDNA, 0.2 µl of specific primer (10µM each), 5 µl of Luna^®^ Universal qPCR Master Mix (New England Biolabs, Ipswich, MA, USA) and 4.3 µl of nuclease-free water. The thermal cycling was performed in triplicate at 94°C for 1 min, followed by 40 cycles of 94°C for 15 s, 65°C for 30 s, and 72°C for 30 s.

### Effect of *Pm*IRF Gene Silencing on Immune-Related Gene Expression

The effect of dsRNA-mediated *Pm*IRF gene silencing on the transcript levels of other immune-related genes was examined by qRT-PCR using primers specific to *P. monodon* antimicrobial peptides (*Pm*PEN3, *Pm*PEN5, ALF*Pm*3, ALF*Pm*6, Crustin*Pm*1, and Crustin*Pm*7) and IFN-like molecules (*Pm*Vago1, *Pm*Vago2, *Pm*Vago3, *Pm*Vago4, and *Pm*Vago5) (**Table 1**). EF1-α served as the internal control for normalization.

### Gene Expression Profiles in Response to Stimulation With Nucleic Acid Mimic

Changes in *Pm*STING, *Pm*IRF, and ALF*Pm*6 transcript levels in *P. monodon* intestine following injection with the nucleic acid mimics poly(dA:dT) and HMW poly(I:C) were evaluated by qRT-PCR. Shrimp (3–5 g) were divided into triplicate groups of 3 shrimps each and 50 µl poly(dA:dT) (2 µg/g) or 50 µl HMW poly(I:C) (2 µg/g) diluted in phosphate-buffered saline [PBS; 137 mM NaCl, 2.7 mM KCl, 4.3 mM Na_2_HPO_4_, and 1.4 mM KH_2_PO_4_ (pH 7.4)] was injected into the second abdominal segment (50 µl per shrimp). The control group was injected with PBS. The shrimps were reared in seawater tanks and the intestine was randomly collected at 0, 3, 6, 24, and 48 h post injection. Total RNA was extracted and first-strand cDNA was synthesized as described above. The RNA from 3 shrimps per treatment at each time point was pooled. qRT-PCR was performed as previously described (30) using target gene-specific primers (**Table 1**). EF1-α was amplified as the internal control and reference standard. Three replicates were prepared for each template with 3 independent replicates for each data point. The Ct value at each time point was normalized to PBS-injected samples. A previously established mathematical model (31) was used to determine the relative expression ratio.

### Cells and Reagents

HEK293T cells were cultured in Dulbecco’s Modified Eagle’s medium (DMEM) (Life Technologies, Carlsbad, CA, USA) with 10% heat-inactivated fetal bovine serum (FBS) (Life Technologies) in an incubator at 5% CO_2_ and 37°C. HMW poly(I:C) and poly(dA:dT) (*In vivo*Gen, San Diego, CA, USA) were separately mixed with Lipofectamine 3000 (Life Technologies) at a 1:1 ratio (µg/µl) in Opti-MEM (Life Technologies) for cell stimulation. Anti-Flag and anti-Myc antibodies were purchased from Sigma-Aldrich (St. Louis, MO, USA).

### Plasmid Construction

Full-length *Pm*STING and *Pm*IRF cDNA sequences were cloned into pFlag-CMV5 (Sigma-Aldrich, St. Louis, MO, USA) and pcDNA3-Myc (Santa Cruz Biotechnology, Santa Cruz, CA, USA) expression plasmids using specific primers (**Table 1**). The 25-µl amplification reaction mixture contained 1× KOD FX PCR buffer, 4 mM dNTP, 0.3 µM each primer, 1 µl normal shrimp cDNA, and 1 U KOD FX DNA polymerase (Toyobo, Osaka, Japan). The PCR thermal cycling conditions were 94°C for 2 min; 35 cycles of 98°C for 10 s, 60°C for 30 s, and 68°C for 1 min 30 s; and 68°C for 7 min. PCR products were separated by agarose gel electrophoresis and bands of the expected size were excised and purified using a FavorPrep GEL/PCR Purification Kit (Favorgen Biotech, Ping-Tung, Taiwan). The purified fragments were cloned into pFlag-CMV5 and pcDNA3-Myc expression plasmids. To construct the pGL3 promoter plasmid (pGL3) harboring ALF*Pm*6 and ALF*Pm*3 promoter sequences, the sequences were amplified by PCR from gill genomic DNA and inserted into the pGL3 plasmid digested with *Bgl*II and *Not*I restriction enzymes. The reporter plasmids for IFN-β and NF-κB were constructed as previously described (32, 33).

### Luciferase Reporter Assay

HEK293T cells (1×10^5^ cell/ml) were cultured in DMEM and seeded in a 24-well plate, then transfected with 100 ng IFN-β or NF-κB reporter plasmid and 500 ng of expression plasmid or empty plasmid using Lipofectamine 3000 in Opti-MEM at a 1:1 ratio (µg/µl). As an internal control, 10 ng of pRL-TK (Promega, Madison, WI, USA) was transfected. After 24 h, cells were stimulated with 1 µg/ml of poly(dA:dT) or HMW poly(I:C) and 6 h later, luciferase activity was detected using the Dual-Glo Luciferase System (Promega, Madison, WI, USA) according to the manufacturer’s instructions, with absorbance measured using a TriStar2 LB 942 Multidetection Microplate Reader (Berthold Technologies, Bad Wildbad, Germany).

### Protein Expression and Co-Immunoprecipitation

HEK293T cells (1×10^6^ cell/ml) were seeded in 10-cm cell culture dishes and transfected with 4 µg of Flag- and Myc-tagged expression constructs using Lipofectamine 3000. After 24 h, the cells were stimulated with 1 µg/ml poly(dA:dT) and HMW poly(I:C) for 6 h and then lysed with homo buffer [150 mM NaCl, 5 mM EDTA (pH 8.0), 25 mM Tris-HCl (pH 8.0), and 0.2% Triton X-100] containing protease inhibitor cocktail (Roche, Basel, Switzerland). After sonication, cell lysates were immunoprecipitated overnight at 4°C with mouse anti-Myc antibody diluted 1:500 and then treated for 4 h at 4°C with protein A sepharose beads (GE Healthcare, Little Chalfont, UK). The beads with immunoprecipitates were washed 3 times with PBS buffer. Whole-cell lysates and immunoprecipitates were probed with the appropriate antibodies.

### Western Blotting

HEK293T cells cultured in 6-well plates were lysed in homo buffer [150 mM NaCl, 5 mM EDTA (pH 8.0), 25 mM Tris-HCl (pH 8.0), and 0.2% Triton X-100] containing protease inhibitor cocktail (Roche). Following centrifugation, the supernatant was mixed with sodium dodecyl sulfate (SDS) sample buffer and proteins were separated by SDS–polyacrylamide gel electrophoresis and transferred to an Immun-Blot polyvinylidene difluoride membrane that was probed with anti-Flag and -Myc antibodies. Protein bands were visualized with horseradish peroxidase (HRP)-conjugated antibodies against mouse, rabbit, or goat IgG (Sigma-Aldrich, St. Louis, MO, USA) using Western Lighting Plus-ECL reagent (Perkin Elmer, Waltham, MA, USA). HRP activity was detected with an LAS 4000 imaging system (Fujitsu Life Sciences, Tokyo, Japan).

### Immunofluorescence Analysis and Confocal Microscopy

Cells were cultured on poly-l-lysine–coated coverslips in 24-well plates for 6 h, then transfected with 500 ng of expression plasmid for 16 h and stimulated with 1 µg/ml of poly(dA:dT) and HMW poly(I:C) for 6 h before fixation with 4% paraformaldehyde for 30 min. The cells were washed 3 times with 0.02% Triton X-100 in PBS, permeabilized with PBS containing 100 mM glycine and 0.2% Triton X-100 for 30 min, blocked overnight at 4°C in PBS containing 10% FBS and 0.02% Triton X-100, and probed overnight at 4°C with anti-Flag and/or -Myc antibody diluted 1:100. The coverslips were then washed and incubated for 1 h at room temperature with Alexa Fluor 488- or Alexa Fluor 568-conjugated anti-mouse and/or -rabbit secondary antibody (both from Invitrogen). Nuclei were stained with Hoechst 33342 (Invitrogen). Stained cells were mounted with Fluoro-KEEPER Antifade Reagent (Nacalai Tesque, Kyoto, Japan), and images were acquired with an LSM 700 laser scanning confocal microscope (Carl Zeiss, Wetzlar, Germany).

### Statistical Analysis

The experiments were performed in three independent experiments with three technical replicates per experiment. Relative gene expression data were obtained according to the method described by Pfaffl and comparisons between groups were analyzed by one-way analysis of variance followed by Duncan’s multiple comparison tests.

## Results

### *Pm*IRF Gene Silencing Inhibits the Expression of Shrimp Antimicrobial Peptides and IFN-Like Molecules

**Figure 1:**
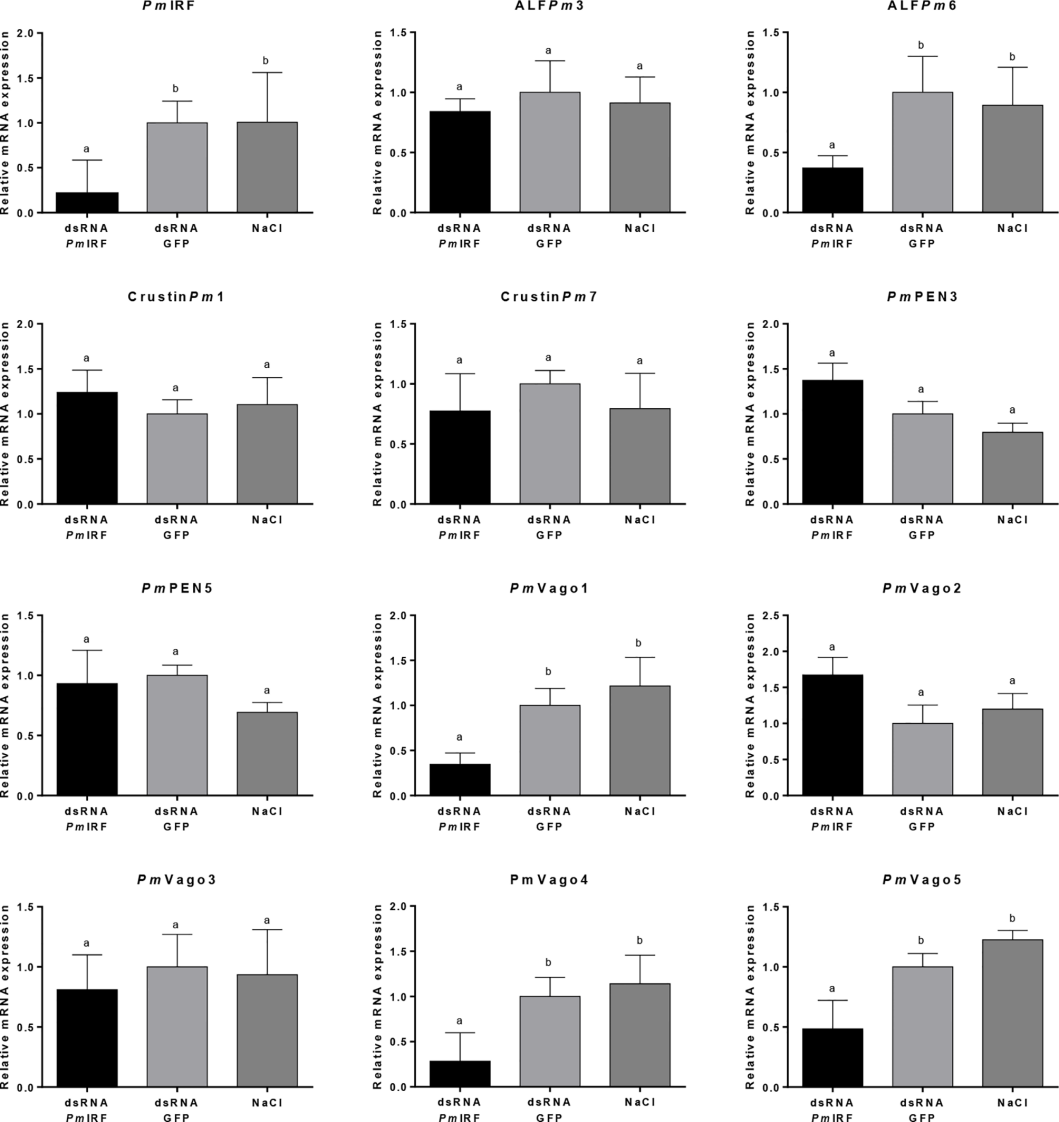
Effect of *PmIRF* knockdown on the expression levels of antiviral and antimicrobial peptide genes. Shrimps were injected with *Pm*IRF dsRNA, *GFP* dsRNA, or 150 mM NaCl. After 24h, the intestine was collected for qRT-PCR analysis. *EF*1*-α* was used as an internal control. Data represent mean ± SD of 3 independently replicated experiments. Significant difference in the mean relative expressions compared with the control group at the level of P < 0.05 is indicated by a different lowercase letter.

IRFs regulate gene expression in both innate and adaptive immunity (34). In order to identify genes that are regulated by *Pm*IRF, we suppressed *Pm*IRF expression by RNA interference (RNAi) and examined the changes in expression of immune-related genes by quantitative real-time (qRT)-PCR. *P. monodon* (3–5 g) was injected with *Pm*IRF dsRNA (5 μg/g shrimp), control green fluorescent protein (GFP) dsRNA, or 150 mM NaCl. Intestine from triplicate groups of shrimp (n = 3 for each group) were collected and extracted the total RNA. *Pm*IRF transcript was depleted by dsRNA-mediated knockdown, whereas injection of GFP dsRNA or NaCl had no effect on *Pm*IRF expression (**Figure 1**). We also analyzed the expression of genes encoding shrimp antimicrobial peptides (*Pm*PEN3, *Pm*PEN5, ALF*Pm*3, ALF*Pm*6, Crustin*Pm*1, and Crustin*Pm*7) and IFN-like molecules (*Pm*Vago1, *Pm*Vago2, *Pm*Vago3, *Pm*Vago4, and *Pm*Vago5) after *Pm*IRF silencing and found that ALF*Pm*6, *Pm*Vago1, *Pm*Vago4, and *Pm*Vago5 were significantly downregulated (p<0.05) compared to the control whereas the expression of other genes (*Pm*PEN3, *Pm*PEN5, ALF*Pm*3, Crustin*Pm*1, Crustin*Pm*7, *Pm*Vago2, and *Pm*Vago3) was unaffected (**Figure 1**). The results suggest that ALF*Pm*6, *Pm*Vago1, *Pm*Vago4, and *Pm*Vago5 are possibly regulated by *Pm*IRF.

### *Pm*IRF Overexpression Activates IFN-β, NF-κB, and ALF*Pm*6 Promoters

To further investigate the function of *Pm*DDX41, *Pm*STING and *Pm*IRF, recombinant of *Pm*DDX41, *Pm*STING and *Pm*IRF proteins were produced in HEK293T cells. The cells were transfected with Flag-tagged-*Pm*DDX41 or Myc-tagged-*Pm*STING or Flag-tagged-*Pm*IRF for 24-72 h. The recombinant protein in HEK293T cells were detected by immunoblotting using anti-Flag or anti-Myc antibody, respectively (**Supplement Figure 1**).

**Figure 2:**
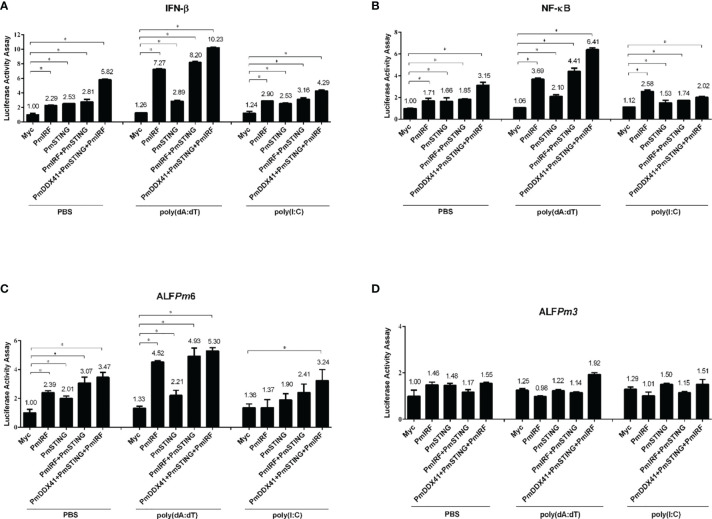
Luciferase assay for promoter activation induced by various immune-related factors in HEK293T cells. **(A–D)** The activation of IFN-β **(A)**, NF-κB **(B)**, ALF*Pm*6 **(C)**, and ALF*Pm*3 **(D)** promoters was evaluated. Cells were co-transfected with 0.5 μg of Flag-tagged *Pm*IRF expression plasmid and 0.5 μg of Myc tagged-*Pm*STING along with IFN-β-Luc, NF-κB-Luc, ALF*Pm*6-Luc, and/or ALF*Pm*3-Luc (all 0.1 μg) plasmid and the Renilla luciferase reporter pRL-TK (0.01 μg) plasmid, then stimulated with poly(dA:dT) or HMW poly(I:C); the luciferase assay was performed after 6 h Data represent mean ± SD of 3 independently replicated experiments. *p < 0.05.

To identify the immune signaling pathway involved in the activation of *Pm*IRF and expression of immune-related genes, we co-transfected a *Pm*IRF overexpression construct and luciferase reporter plasmid driven by the IFN-β, NF-κB, ALF*Pm*3, or ALF*Pm*6 promoter into human embryonic kidney (HEK293T) cells. In PBS-control condition, *Pm*IRF overexpression increased the activity of the IFN-β, NF-κB, and ALF*Pm*6 promoters 2.29, 1.17 and 2.39 fold, respectively; and in cells stimulated with the nucleic acid mimic deoxyadenylic–deoxythymidylic acid sodium salt [poly(dA:dT)], promoter activity was increased 7.27, 3.69, and 4.52 fold, respectively (**Figures 2A–C**) while poly(I:C) induced the promoter activity of IFN-β, NF-κB and ALF*Pm*6 to a similar extent to the PBS treatment. ALF*Pm*3 promoter activity was unchanged by stimulation with DNA mimics compared to the control (**Figure 2D**).

To examine the function of *Pm*IRF in the cytosolic nucleic acid sensing pathway, we co-transfected *Pm*STING and *Pm*IRF expression plasmids into HEK293T cells. *Pm*STING overexpression induced the activation of IFN-β, NF-κB, and ALF*Pm*6 promoters 2.53, 1.66, and 2.01-fold, respectively; in the presence of poly(dA:dT), the activity was induced 2.89, 2.10, and 2.21 fold, respectively, as determined with the luciferase assay (all p<0.05). Similarly, co-expression of *Pm*STING and *Pm*IRF increased IFN-β, NF-κB, and ALF*Pm*6 promoter activity 2.81, 1.85, and 3.07-fold, respectively; and stimulation with poly(dA:dT) enhanced the activity 8.20, 4.41, and 4.93 fold, respectively. Notably, co-expression of *Pm*DDX41, *Pm*STING, and *Pm*IRF synergistically increased IFN-β, NF-κB, and ALF*Pm*6 promoter activity 5.82, 3.15, and 3.47-fold, respectively, without stimulation and 10.23, 6.41, and 5.30 fold, respectively, in the presence of poly(dA:dT) (**Figures 2A–C**). These results suggest that *Pm*IRF is involved in the STING-dependent cytosolic DNA sensing pathway leading to interferon and AMPs activation.

### DNA Sensing Pathway Genes Are Induced in *P. monodon* Intestine Following Stimulation With Nucleic Acid Mimic

**Figure 3:**
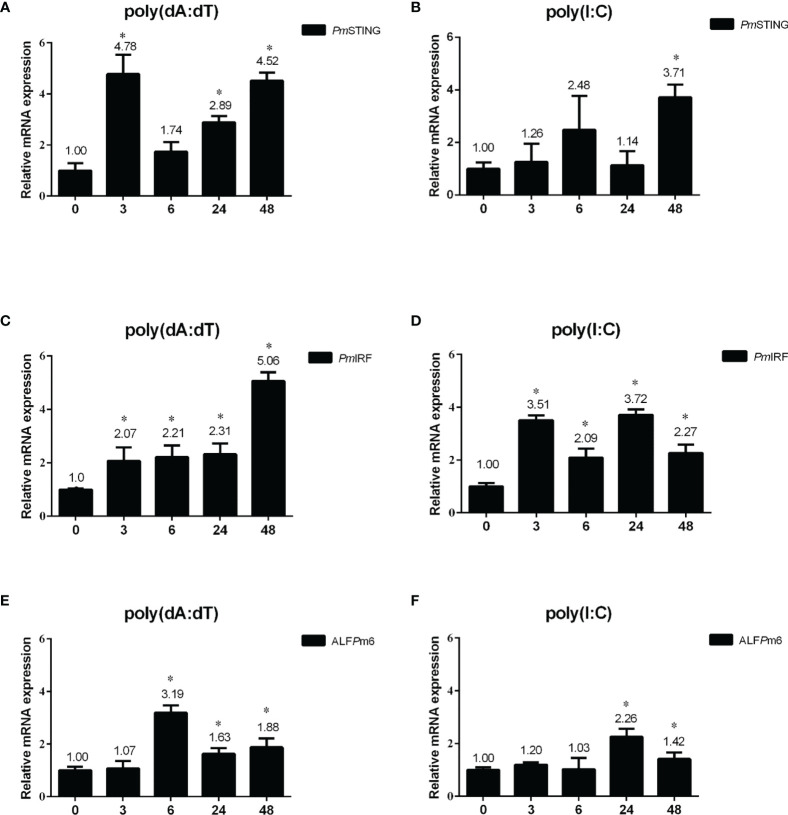
Expression profiles of immune-related genes in *P. monodon* intestine after injection of nucleic acid mimics. **(A–F)** Relative expression levels of *Pm*STING **(A, B)**, *Pm*IRF **(C, D)**, and ALF*Pm*6 **(E, F)** were evaluated by qRT-PCR at 0, 3, 6, 24, and 48 h after injection of poly(dA:dT) or HMW poly(I:C), with the *EF1-α* gene serving as an internal control. The expression level at 0 h was set as the baseline (1.0). Data represent mean ± SD of the assay performed with triplicate samples. *p < 0.05.

We investigated whether nucleic acid mimics could also activate DNA sensing pathway-related genes (*Pm*STING, *Pm*IRF, and ALF*Pm*6) in shrimp intestine by qRT-PCR. After poly(dA:dT) injection, *PmSTING* expression was significantly upregulated at 3, 24, and 48 h post injection (p<0.05), with the greatest increase (4.78 fold) at 3 h (**Figure 3A**). HMW poly(I:C) also induced *Pm*STING expression 3.71 fold at 48 h (p<0.05; **Figure 3B**). *Pm*IRF transcript was upregulated 2.07, 2.21, 2.31, and 5.06 fold at 3, 6, 24, and 48 h, respectively, after poly(dA:dT) injection (**Figure 3C**); and HMW poly(I:C) injection induced *Pm*IRF expression 3.51, 2.09, 3.72, and 2.27 fold at 3, 6, 24, and 48 h, respectively (**Figure 3D**). Moreover, after poly(dA:dT) injection, ALF*Pm*6 expression was increased 3.19, 1.63, and 1.88 fold at 6, 24, and 48 h, respectively (**Figure 3E**). HMW poly(I:C) injection also enhanced ALF*Pm*6 expression 2.26 and 1.42 fold at 24 and 48 h, respectively (both p<0.05; **Figure 3F**). Thus, the expression of *Pm*STING, *Pm*IRF, and ALF*Pm*6 was induced by both nucleic acid mimics.

### Interaction of *Pm*IRF and *Pm*STING in HEK293T Cells

**Figure 4:**
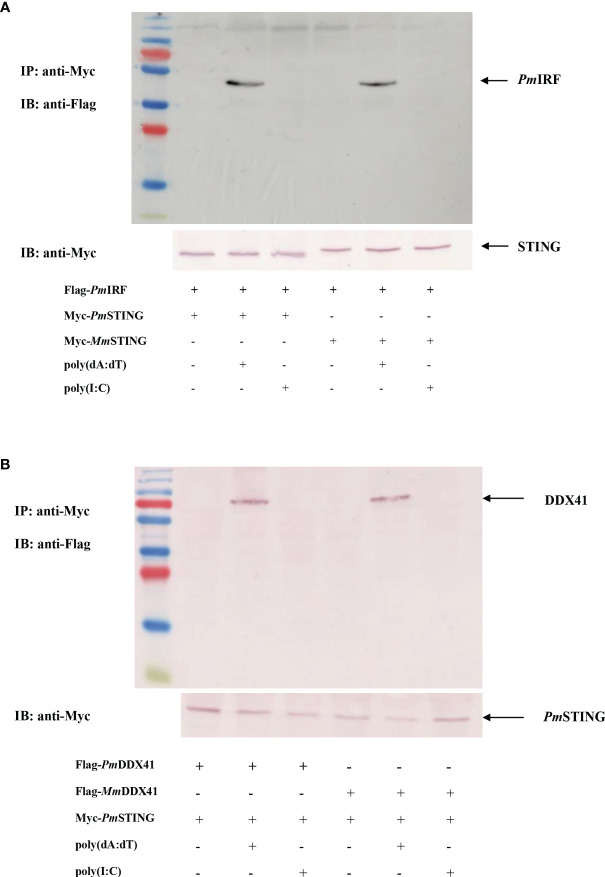
Co-immunoprecipitation of *P. monodon* proteins in HEK293T cells. **(A, B)** Cells were co-transfected with Flag-tagged full-length *Pm*IRF **(A)** or *Pm*DDX41 **(B)** plasmid, and 24 h later the co-immunoprecipitation of the *P. monodon* proteins with Myc-tagged *Pm*STING was detected following stimulation with poly(dA:dT) or HMW poly(I:C) for 6 h using HRP-conjugated anti-Flag and -Myc antibodies; the mouse homologs *Mm*STING [in panel **(A)**] and *Mm*DDX41 [in panel **(B)**] were used as controls.

To further clarify the function of *Pm*IRF in the STING-dependent cytosolic DNA sensing pathway, we analyzed the interaction between *Pm*IRF and *Pm*STING. We co-transfected HEK293T cells with plasmids encoding Flag-tagged *Pm*IRF and Myc-tagged *Pm*STING; a plasmid encoding Myc-tagged *Mus musculus* (*Mm*STING) protein served as a control. The proteins were incubated with anti-Myc antibody conjugated with A-sepharose beads and then detected by western blotting using anti-Flag or -Myc antibody. *Pm*IRF bound *Pm*STING after stimulation with poly(dA:dT) but not poly(I:C) (**Figure 4A**).

*Pm*DDX41 was shown to bind mouse STING protein upon poly(dA:dT) stimulation in HEK293T cells (27). We recently identified a *P. monodon* homolog of STING (*Pm*STING) (20). As some amino acid residues of *Pm*STING are conserved from arthropods to mammals, we speculated that *Pm*STING could bind to cyclic dinucleotides in a manner similar to mammalian STING (19). To test this hypothesis, we carried out a co-immunoprecipitation assay to analyze the interaction of *Pm*DDX41 and *Pm*STING in HEK293T cells. The cells were co-transfected with plasmids encoding Flag-tagged *Pm*DDX41 and Myc-tagged *Pm*STING, with Flag-tagged mouse *Mm*DDX41 recombinant protein used as a control. Cell lysates were precipitated with an anti-Myc antibody conjugated with protein A-sepharose beads and the Flag- or Myc-tagged protein was detected by western blotting using an anti-Flag or -Myc antibody, respectively. *Pm*DDX41 was found to interact with *Pm*STING after poly(dA:dT) but not HMW poly(I:C) stimulation (**Figure 4B**). Additionally, *Mm*DDX41 interacted with *Pm*STING in the presence of poly(dA:dT) (**Figure 4B**). These results confirm that STING mediates cytosolic DNA sensing in response to a signal from DDX41 that activates IRF, leading to the production of IFN-like molecules.

### Subcellular Localization of *Pm*IRF, *Pm*STING, and *Pm*DDX41 in HEK293T Cells

**Figure 5:**
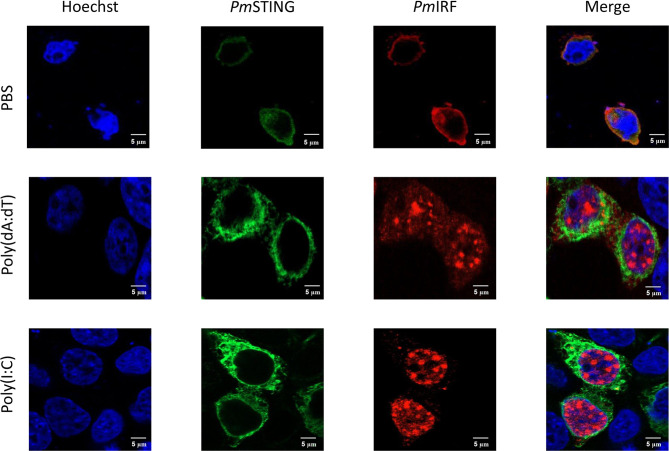
Subcellular localization of *Pm*IRF and *Pm*STING in HEK293T cells. Cells were co-transfected with Flag-tagged *Pm*IRF and Myc-tagged *Pm*STING expression plasmids for 24 h, then stimulated with 1 μg/ml poly(dA:dT) or HMW poly(I:C) for 6 h before labeling with an Alexa Fluor 568-conjugated anti-Flag antibody (red) and Alexa Fluor 488-conjugated anti-Myc antibody (green). Nuclei were stained with Hoechst 33342 (blue). Scale bar, 5 μm. Fluorescence was detected by laser scanning confocal microscopy (63× magnification).

**Figure 6:**
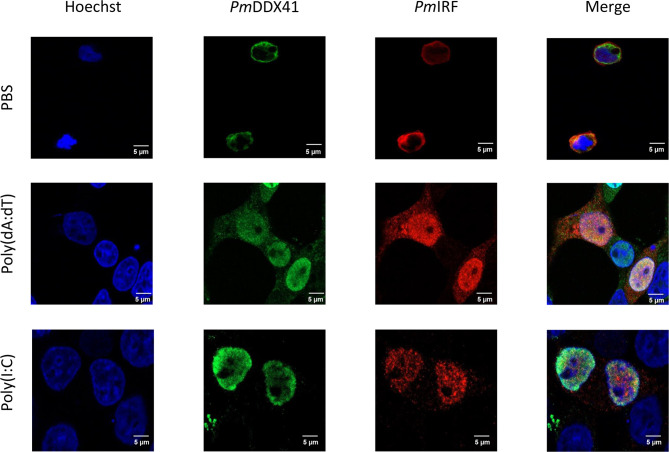
Subcellular localization of *Pm*IRF and *Pm*DDX41 in HEK293T cells. Cells were co-transfected with Myc-tagged *Pm*DDX41 and Flag-tagged *Pm*IRF expression plasmids for 24 h, then stimulated with 1 μg/ml poly((dA:dT) or HMW poly(I:C) for 6 h before labeling with an Alexa Fluor 568-conjugated anti-Flag antibody (red) and Alexa Fluor 488-conjugated anti-Myc antibody (green). Nuclei were stained with Hoechst 33342 (blue). Scale bar, 5 μm. Fluorescence was detected by laser scanning confocal microscopy (63× magnification).

In the STING-dependent cytosolic DNA sensing pathway, DDX41 interacts with viral dsDNA and STING, leading to activation of TBK1 or IKK and IFN production *via* IRF. To clarify the mechanism by which *Pm*IRF senses nucleic acids, we performed immunofluorescence microscopy to examine the subcellular localization of *Pm*IRF and *Pm*STING in HEK293T cells co-transfected with Myc-tagged *Pm*STING and Flag-tagged *Pm*IRF expression plasmids and stimulated 24 h later with poly(dA:dT) or HMW poly(I:C). *Pm*IRF and *Pm*STING were localized in the cytoplasm in unstimulated cells (**Figure 5**). After treatment with poly(dA:dT) and HMW poly(I:C), *Pm*IRF was detected in the cytoplasm and nucleus whereas *Pm*STING remained exclusively cytoplasmic (**Figure 5**). In cells co-transfected with Myc-tagged *Pm*DDX41 and Flag-tagged *Pm*IRF plasmids, *Pm*IRF co-localized with *Pm*DDX41 in the cytoplasm. Poly(dA:dT) and HMW poly(I:C) treatment induced the expression of *Pm*IRF and *Pm*DDX41, which were localized in both the cytoplasm and nucleus (**Figure 6**).

## Discussion

IRFs participate in pathogen-induced innate and acquired immunity in both vertebrates and invertebrates by regulating the expression of genes in multiple signaling pathways, especially those involved in antiviral immunity and that control cell differentiation and growth, apoptosis, the DNA damage response, and tumor suppression (22, 34, 35). To date, 9 IRFs have been identified in mammals; these play critical roles in the activation of immune responses (34, 36). The first crustacean IRF-like gene was identified in Pacific white shrimp; subsequent analyses revealed that *Lv*IRF is involved in antiviral immunity, similar to the mammalian homologs. We recently identified *Pm*IRF and *Pm*STING in *P. monodon* and demonstrated through RNAi-mediated loss-of-function experiments that they contribute to antiviral defense in shrimp (20).

The cytosolic DNA sensing pathway plays an important role in host defense. Upon infection with a virus, host PRRs sense viral PAMPs and activate an immune response. cGAS is one of the cytosolic DNA sensor in the innate immune system (12). It detects cytosolic DNA from intracellular bacteria, damaged mitochondria, DNA viruses and retroviruses and triggers IFNs response (37, 38). Moreover, DDX41 is also a DNA-binding protein that can detect viral or bacterial DNA and activates intracellular signaling cascades of the innate immune system (10, 39, 40). DDX41 was reported to activate the endoplasmic reticulum (ER) adaptor protein STING (10, 41), and various DNA viruses were shown to induce STING signaling. Recently, *Pm*DDX41, *Pm*STING, and *Pm*IRF were identified in the shrimp *P. monodon* and were shown to be involved in the innate immune response against WSSV infection (27). In this study, we further investigated the regulation of *Pm*IRF *via* the STING-dependent cytosolic DNA sensing pathway. Silencing the *Pm*IRF gene reduced the expression of several antimicrobial peptides and IFN-like molecules in shrimp. In our previous work, depletion of *Pm*DDX41 by RNAi increased the mortality rate of WSSV-infected shrimp and significantly reduced the mRNA expression levels of several immune-related genes (*Pm*IKKβ, *Pm*IKKϵ, *Pm*Relish, *Pm*Cactus, *Pm*Dorsal, *Pm*PEN3, *Pm*PEN5, and ALF*Pm*6) (42). Moreover, the expression levels of IFN-like molecules (*Lv*Vago4 and *Lv*Vago5) were significantly decreased in IRF-silenced *L. vannamei* (26). Vago is a viral infection-inducible peptide first identified in *Drosophila* that can suppress the viral load of *Drosophila* C virus in the fat body (43). In mosquito, Vago is a secreted peptide that blocks viral infection by activating JAK–STAT signaling, which is homologous to the mammalian IFN system (44). Thus, ALF*Pm*6, *Pm*Vago1, *Pm*Vago4, and *Pm*Vago5 are likely target genes of *Pm*IRF in the immune signaling pathway of *P. monodon*.

The results of the promoter activity assay demonstrated that *Pm*IRF activated the IFN-β, NF-κB, and ALF*Pm*6 promoters in HEK293T cells, especially upon stimulation with nucleic acid mimics. DDX41 and STING overexpression synergistically enhanced the activity of the IFN-β promoter in L929 mouse fibroblast cells (10). In *P. monodon*, co-transfection of *Pm*DDX41 and *Mm*STING plasmids enhanced the promoter activity of IFN-β and NF-κB (27). In *L. vannamei*, IRF activated promoters which contain ISRE element to regulate the expression of mammalian type I IFNs and induce an antiviral state in S2 cells (26). Our results indicated that *Pm*IRF is a downstream gene in the *Pm*DDX41–*Pm*STING DNA sensing pathway that regulates the activity of IFN-β, NF-κB, and ALF*Pm*6 promoters.

We demonstrated that *Pm*DDX41, *Pm*STING, and *Pm*IRF activated the promoter of the ALF*Pm*6 gene but not the ALF*Pm*3 gene. In a previous study, *Pm*DDX41 knockdown resulted in the downregulation of ALF*Pm*6 expression (42). On the contrary, *Pm*STING, *Pm*IRF, and ALF*Pm*6 levels was strongly upregulated by stimulation with nucleic acid mimic [poly(dA:dT) and to a lesser extent, HMW poly(I:C)]. *Pm*DDX41, a DNA sensor, was previously shown to be upregulated upon infection with DNA virus and stimulation with nucleic acid mimic (27, 42). In one study, DDX41 recognized a dsDNA virus in vertebrates and acted through the STING–TBK1–IRF3 pathway to directly bind DNA and STING *via* its DEAD box domain (10). In *L. vannamei* infected with WSSV or injected with poly(I:C), IRF was shown to be upregulated in the hepatopancreas (26); and *Pm*IRF and ALF*Pm*6 transcripts were upregulated in *P. monodon* challenged with WSSV (20, 26, 45). Thus, *Pm*STING, *Pm*IRF, and ALF*Pm*6 respond more specifically to DNA virus or mimic than to RNA virus or mimic, and may be involved in the nucleic acid-induced antiviral immune response in shrimp.

IRFs have a conserved N-terminal region of about 100 amino acid residues, including 5 conserved Tryptophan that mediate DNA binding (46). Here we found that *Pm*IRF interacted with *Pm*STING in HEK293T cells following stimulation with poly(dA:dT) but not HMW poly(I:C). STING Ser366 participates in IRF3 binding and activation, and its mutation to alanine (S366A) abolished DNA-induced IRF3 activation (47). The amino acid sequence of *Pm*STING was analyzed and found the ‘PLPLRT/SD’ motif which might also contribute to the interaction between *Pm*STING and *Pm*IRF (20). However, the investigation of crucial domain responsible for the function of *Pm*STING will be further performed. We also found that *Pm*IRF co-localized with *Pm*STING in the cytoplasm but was translocated to the nucleus while *Pm*STING remained cytoplasmic upon treatment with HMW poly(dA:dT) and poly(I:C). In *L. vannamei*, *Lv*IRF protein is mainly present in the cytoplasm but is translocated to the nucleus after WSSV infection or poly(I:C) treatment (26). Moreover, *Pm*STING bound *Pm*DDX41 in the presence of DNA mimic and the two proteins were co-localized in the cytoplasm, but the latter was translocated to the nucleus while *Pm*STING remained cytoplasmic following the stimulation. Similar results were observed in our previous study in HEK293T cells co-transfected with *Pm*DDX41 and *Mm*STING plasmids and stimulated with poly(dA:dT) (27), as well as in *Danio rerio* (48). In vertebrates, the DDX41–STING complex was shown to localize in the cytosol, and poly(dA:dT) stimulation reduced the expression of DDX41 and STING in the ER and mitochondria (10). STING was translocated along with TBK1 from the ER to the endosome in murine embryonic fibroblasts (49). Thus, *Pm*DDX41 may function as a DNA sensor in the cytosol and interacts with *Pm*STING to form a complex with TBK and *Pm*IRF that enters the nucleus and activates IFN and other genes related to the antiviral response.

TLRs activate the production of type I IFNs through IRFs (2). Signaling through TLRs can be divided into TIR domain-containing adapter-inducing IFN-β (TRIF)- and MyD88-dependent pathways (2, 50). In the latter, IRF4, IRF5, and IRF7 directly interact with MyD88 to regulate the expression of immune-related genes. IRF7 is essential for type I IFN gene induction by TLR7 or TLR9; IRF5 is required for the expression of pro-inflammatory cytokine genes (50–52); and IRF3 plays an essential role in the TRIF-dependent induction of type I IFN genes by TLR3 and TLR4 *via* TBK1 (2).

Poly(dA:dT) and poly(I:C) are the synthetic compounds which represent as a DNA virus and RNA virus., respectively. Both of them are potent inducers of the innate antiviral response in vertebrates. Poly(dA:dT) is recognized by cytosolic DNA sensors (CDS), including cGAS, AIM2, DAI, DDX41, IFI16, and LRRFIP1, triggers the production of type I interferons (10, 12, 53). Moreover, it is sensed to the cytosolic DNA sensor AIM2 triggers the formation of an inflammasome and the subsequent secretion of IL-1β and IL-18 (54). Poly(I:C) is recognized by TLR3 which mediated the IFNs synthesis (55). These signaling pathways shared the immune-related protein such as STING, IKK, TBK1, and IRF3. After poly(dA:dT) stimulation, *Pm*STING which composed of c-di-GMP-binding domain (CBD) at C-terminal (20), directly binds to the DNA mimic virus and sends the signal to activate *Pm*IRF leading to IFNs production. *Pm*IRF is the downstream gene in the signaling cascade so, poly(dA:dT) and poly(I:C) might induce *Pm*IRF transcript.

**Figure 7:**
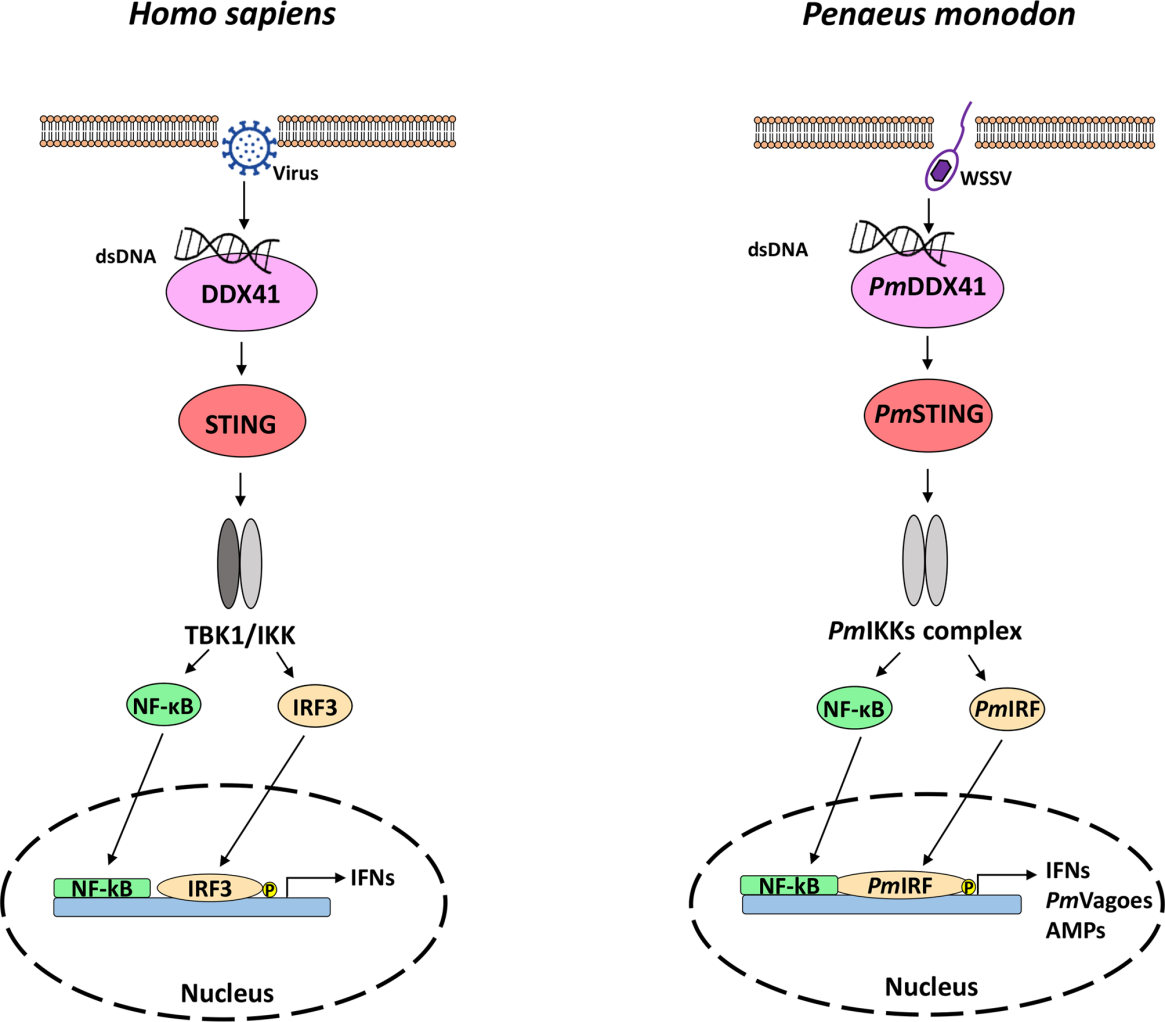
The DNA sensing pathway in mammals (left) and shrimp (right). The homologs of mammalian genes were found in shrimp, indicating that the signaling pathway is conserved from crustaceans to mammals. In mammals, dsDNA is recognized by DDX41, which induces IFN production [adapted from ref. (57)]. In the present study, we showed that dsDNA released from a viral pathogen such as WSSV interacts with the cytosolic DNA sensor *Pm*DDX41 to induce *Pm*STING, thereby promoting the translocation of *Pm*IRF to the nucleus, resulting in the production of IFNs and antimicrobial peptides that eliminate the viral infection.

Together with previous findings on the components of the cytosolic DNA sensing pathway in shrimp (*Pm*DDX41, *Pm*STING, *Pm*IKKs, *Pm*IRF, and *Pm*Vago) (20, 42, 56), we propose a model of how these proteins interact in the cytosolic DNA sensing pathway to activate the antiviral immune response in *P. monodon* (**Figure 7**). Possibly during infection with the DNA virus WSSV, dsDNA is detected and bound by the DNA sensor *Pm*DDX41, which forms a complex with *Pm*STING that acts *via* TBK–IKK–IRF3 to induce the IFN response. This research extends our knowledge of the regulatory role *Pm*IRF in the antiviral response of crustaceans, and provides insight into the molecular mechanism of the cytosolic DNA sensing pathway in *P. monodon*.

## Data Availability Statement

The original contributions presented in the study are included in the article/**Supplementary Material**. Further inquiries can be directed to the corresponding author.

## Author Contributions

AT and PA contributed to the experimental design and helped to obtain funding. TK conceived the study with HEK293T cells. SS performed the experiments and wrote the manuscript. AT, TK, and PA reviewed and edited the manuscript. All authors contributed to the article and approved the submitted version.

## Funding

This work was supported by a grant from the Thailand Research Fund (International Research Network) (Scholar no. IRN61W0001 to AT) and The Second Century Fund (C2F), Chulalongkorn University (to SS). We also gratefully acknowledge an additional support from the Ratchadaphisek Somphot Endowment Fund, Chulalongkorn University to the Center of Excellence for Molecular Biology and Genomics of Shrimp.

## Conflict of Interest

The authors declare that the research was conducted in the absence of any commercial or financial relationships that could be construed as a potential conflict of interest.

## Publisher’s Note

All claims expressed in this article are solely those of the authors and do not necessarily represent those of their affiliated organizations, or those of the publisher, the editors and the reviewers. Any product that may be evaluated in this article, or claim that may be made by its manufacturer, is not guaranteed or endorsed by the publisher.
