# Supplementary material for: Penaeus monodon Interferon Regulatory Factor (PmIRF) Activates IFNs and Antimicrobial Peptide Expression via a STING-Dependent DNA Sensing Pathway
Source: Front Immunol. 2022 Jan 10;12:818267. doi: 10.3389/fimmu.2021.818267 (PMC8784814; doi:10.3389/fimmu.2021.818267)
Supplement: Supplementary file 1 [file DataSheet_1.docx]

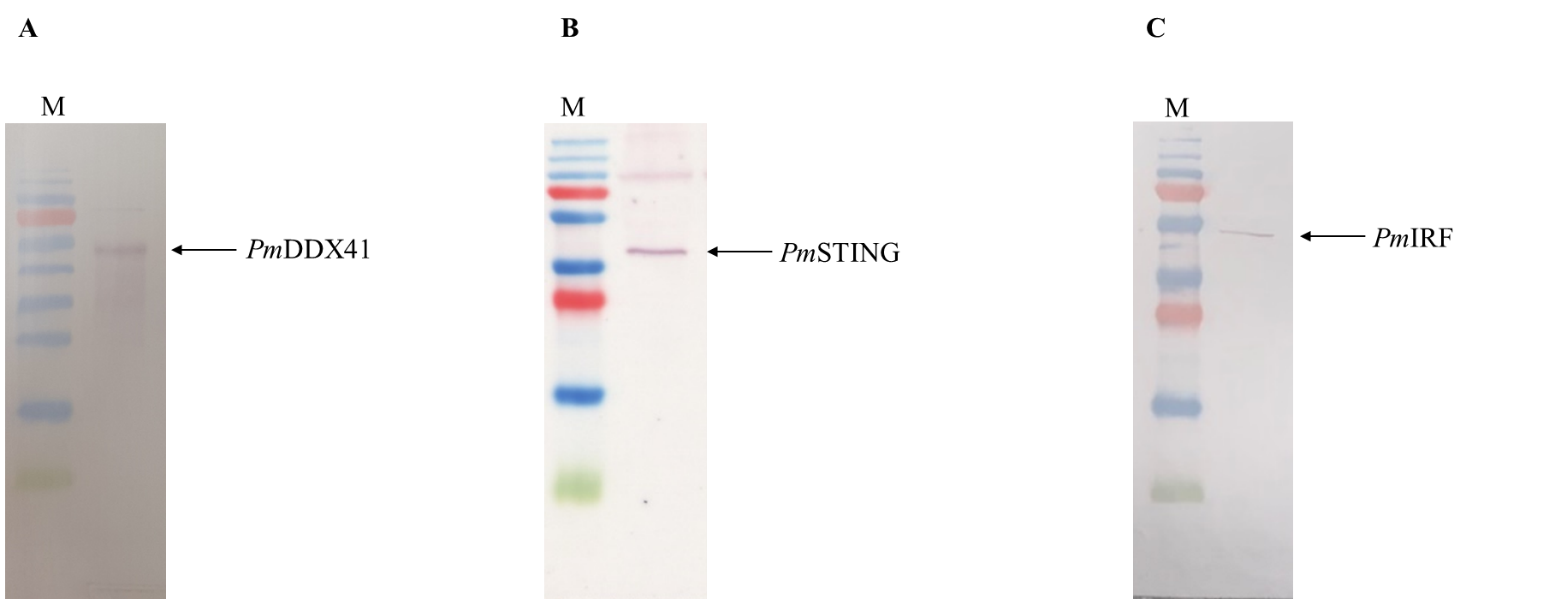


**Supplement Figure 1** The western blotting of *Pm*DDX41, *Pm*STING and *Pm*IRF proteins. Overexpression of *Pm*DDX41 (A), *Pm*STING (B) and *Pm*IRF (C) protein in HEK293T cells which were transfected with 4 µg of recombinant plasmid encoding Flag or Myc tags. After 24h, cells were harvested and sonicated. *Pm*DDX41, *Pm*STING and *Pm*IRF proteins were separated by centrifugation and analyzed by SDS-PAGE. Immunoblotting was carried out with anti–Flag (Sigma), and anti–Myc (Sigma) antibodies. Lane M indicated the Prestained protein marker.
